# Determining the Feasibility of a Cadmium Exposure Model to Activate the Inflammatory Arm of PANoptosis in Murine Monocytes

**DOI:** 10.3390/ijms251910339

**Published:** 2024-09-26

**Authors:** Samuel Camilli, Tanush Madavarapu, Ritaj El Ghissassi, Apoorva Bhargavi Desaraju, Carli Busler, Ramani Soundararajan, Brenda Flam, Richard Lockey, Narasaiah Kolliputi

**Affiliations:** Internal Medicine, Allergy and Immunology, University of South Florida, Tampa, FL 33620, USA; scamilli@bu.edu (S.C.); tanushm@usf.edu (T.M.); ramanis@usf.edu (R.S.); bflam@usf.edu (B.F.); rlockey@usf.edu (R.L.)

**Keywords:** cadmium exposure, PANoptosis, murine, monocytes

## Abstract

A prevalence of cigarette smoking can cause the accumulation of cadmium (Cd^2+^) in the lungs, kidneys, and blood. The effects of exposure can cause multiple chronic disease types to emerge in the affected organ systems. The only moderately effective therapeutic option is chelation therapy; the health risks associated with this therapy have caused much criticism. The disease types associated with Cd^2+^ toxicity have inflammatory components and greatly impact innate immunity. These factors are affected at the cellular level and cause pathways like apoptosis, pyroptosis, and necroptosis. A development in understanding these pathways stipulates that these three pathways act as one complex of pathways, known together as PANoptosis. The inflammatory mechanisms of PANoptosis are particularly interesting in Cd^2+^ toxicity due to its inflammatory effects. Proteins in the gasdermin family act to release inflammatory cytokines, like interleukin-1β, into the extracellular environment. Cytokines cause inflammatory disease pathologies like fibrosis and cancer. RAW 264.7 monocytes are key in the murine immune system and provide an excellent model to investigate Cd^2+^ toxicity. Exposure of 0–15 µM CdCl_2_ was sufficient to increase expression of cleaved gasdermin D (GSDMD) and gasdermin E (GSDME) in this cell type. Cd^2+^ also exhibits a dose–dependent cytotoxicity in this cell type.

## 1. Introduction

Since the 19th century, heavy metals such as lead, mercury, and arsenic have been known to be dangerous to human health [1,2]. Primarily, these heavy metals act to disrupt normal cellular function, resulting in dysfunctional body systems and disease. The most common exposure routes are physical consumption, but, as industrial ventures have developed, other exposure routes have become prevalent.

Cadmium (Cd^2+^) is a transition metal that is highly hazardous to human health. Additionally, Cd^2+^ cannot be metabolized in the body and is particularly difficult to clear due to a 10–35year half-life [3]. The most direct route of exposure for humans is cigarette smoke inhalation. Each cigarette contains about 1–2 µg of Cd^2+^ [4]. Chronic diseases like chronic obstructive pulmonary disease (COPD), emphysema, and cancer are associated with prolonged cadmium exposure [4,5]. For nonsmokers, Cd^2+^ exposure is most common through the food chain, with leafy greens, peanuts, soybeans, potatoes, grains, and sunflower seeds having the highest levels of Cd^2+^ (~0.05–0.12 mg/kg) [6]. Cd^2+^ is taken up by plants through polluted soils, generally from Cd^2+^ generated through the burning of fossil fuels or municipal waste [6]. In multiple countries, it has been found that Cd levels in humans are well above the safe level for many organ systems [7], and the risk of chronic disease has risen as a result [8]. Exposure to Cd is associated with cardiovascular alterations and the development of osteoporosis, rheumatoid arthritis (RA), and osteoarthritis (OA) [9,10]. The mounting prevalence of toxic Cd^2+^ poses a critical public health concern, and effective therapies have yet to be discovered. Currently, the most prominent method of clearing heavy metals from the body is chelation. The chelating agent will bind to metal ions in the blood and form a complex that can be more easily excreted [11]. However, these chelators are only able to clear a low concentration of metal ions and are subject to competitive binding by other metals. Some chelating agents are highly specific to a certain metal ion, but others that have multiple targets could inadvertently remove beneficial metal ions from the blood (Ca^2+^, Na^+^, Zn^2+^).

At a cellular level, Cd^2+^ toxicity is reported to be deleterious to mitochondrial function, innate immune regulation, and the fate of the cell overall. Mitochondrial dysfunction occurs primarily through the production of reactive oxygen species (ROS) that contribute to the development of a multitude of diseases [12]. Cd^2+^ generates ROS by inducing oxidative stress in cells, thereby disrupting the balance between ROS production and antioxidant defense mechanisms in cells. This leads to ROS accumulation and oxidative damage [13,14], which can interfere with vital processes like the electron transport chain or alter the mitochondrial membrane potential. Insummary, the damage caused by Cd^2+^-mediated ROS can lead to a host of diseases, such as cancer and other cardiovascular and neurodegenerative diseases [15,16]. Cd^2+^ can also cause oxidative stress and inflammation in the lungs [13]. This can lead to COPD, asthma, and lung cancer [17].

Enough ROS production via Cd^2+^ toxicity induces programmed cell death [18]. This type of programmed cell death is known as apoptosis, which is a well-established mechanism in which the cell lyses and “dies” in response to endogenous or exogenous stimuli. Many diseases result from apoptosis, whether they are due to excessive or insufficient cell death [19]. A primary example of a disease caused by apoptosis would be cancer. Cancer cells can avoid apoptosis through the help of mutations in apoptotic regulatory genes or even through upregulating anti-apoptotic proteins [20,21]. 

A more recent discovery in cell death modulation has revealed a highly inflammatory pathway called pyroptosis [22]. In the innate and adaptive immune system, pyroptosis can be beneficial in regulating cell homeostasis and clearing pathogens via the release of inflammatory cytokines [23,24]. Thus, aberrant pyroptosis and subsequent overexpression of inflammatory cytokines can lead to tumor progression and accelerate disease development [25,26]. One characteristic of pyroptosis that makes it unique from other forms of cell death is the change in cellular shape that results. This process is initiated mainly by caspase proteins, specifically caspase-1. However, recent findings support the cleavage of gasdermin D (GSDMD) as the committed step of pyroptosis [27]. After GSDMD is cleaved at the N-terminal, it can oligomerize and embed in the cell membrane. Once the pore is formed, active cytokines like IL-1β can diffuse to the extracellular environment and water can begin to enter the cell. The osmotic pressure of the cell is drastically increased until the membrane begins to bleb, swell, and burst. With the subsequent inflammatory response initiated by cytokine storm, it seems logical to understand cadmium’s role in pyroptosis. Fortunately, there are multiple reports that implicate Cd^2+^ in pyroptosis in renal tubular epithelial cells of ducks [28,29], testicular tissue of mice [30], and triple–negative breast cancer cells [31]. However, these reports fail to build an understanding of how Cd^2+^ impairs the activity of the immune system. Thus, pyroptosis needs to be established in key immune regulators, such as macrophages. 

Macrophages are a type of phagocyte that can engulf and break down foreign substances detected in the body and, when activated, become either M1 or M2 macrophages [32]. The macrophages of interest for our work are RAW 264.7 macrophages, which are inactive (M0). This cell model has been used in many studies of inflammatory disease and recently has been used in the implication of cadmium toxicity in osteoclast differentiation and as a contributor to atherosclerosis [33,34,35]. Murine macrophages are an excellent model to represent disease pathogenesis and provide foundational evidence that can be developed to conduct human studies. Cadmium’s effect on the immune system may contribute to its role in multiple disease types across different body systems, but these mechanisms remain elusive. In the context of inflammatory cell death and immune homeostasis, some very intriguing interactions have been discovered. 

The interplay between Cd^2+^ and cellular immune response is of great interest, as it has the potential to provide a mechanistic understanding of disease outcomes and reveal novel targets for therapeutic intervention. The purpose of this study was to identify the effects of Cd^2+^ exposure on the inflammatory component of PANoptosis in the innate immune response.

## 2. Results

### 2.1. Cadmium Toxicity Is Dose-Dependent in RAW 264.7 Murine Macrophages 

**Figure 1:**
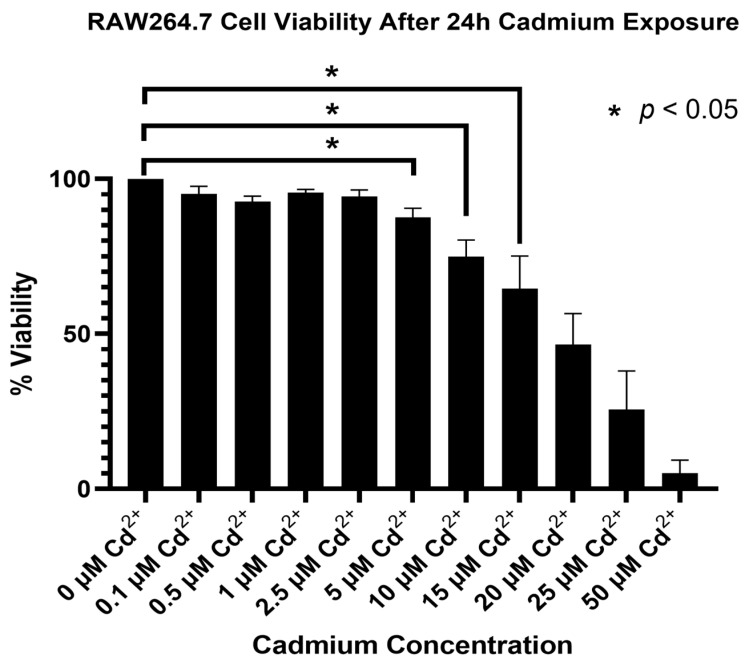
Establishing a viable Cd^2+^ exposure concentration for in vivo study. A cytotoxicity assay (GLPBio, GK10001) was performed on RAW264.7 macrophages plated to a 96-well microplate. The cells were exposed to varying concentrations of CdCl_2_ (0–50 μM) for 24 h and incubated with CCK8 solution per the manufacturer’s instructions. The absorbance of the plate was read at 450 nm. The viability of cells exposed to 5 μM, 10 μM, and 15 μM CdCl_2_ was significantly less than that of controls (*p* = 0.027, 0.021, 0.039 respectively, *n* = 3).

The cells exposed to 5 µM, 10 µM, and 15 µM CdCl_2_ showed significant decreases in viability as compared to control cells exposed to no CdCl_2_ (Figure 1, *p* < 0.05). Not only were they significantly less than the controls, but the mean values for cell viability were also sufficient to observe the inflammatory cell death that is pertinent to this investigation. The LD 50 in these cells was calculated to be 12.2 µM CdCl_2_. Too large a number of live cells may not reveal the true mechanisms of cadmium toxicity that we are interested in, while too few live cells will not allow for observations of the mechanisms of toxicity in process, rather, only its aftereffect. Therefore, we selected the three concentrations of CdCl_2_ (5, 10, and 15 µM) as our experimental groups for evaluating protein expression and activity during Cd^2+^ toxicity.

### 2.2. GSDME and GSDMD Are Cleaved during Cd Exposure

**Figure 2:**
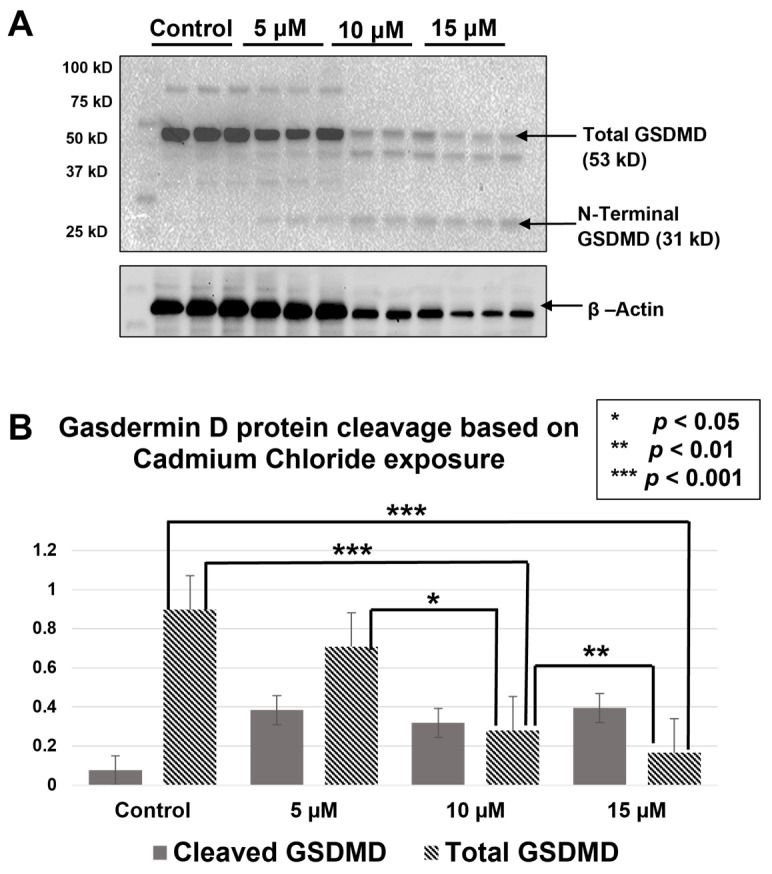
Western blot analysis of gasdermin D. (**A**) A total of 10 μg of whole cell lysate was separated on 10% SDS-PAGE. The blot was probed with GSDMD antibody (abcam, 1:1000). The blot was developed with ECL. β-Actin was used as a loading control. (**B**) Band intensity was determined using ImageJ, and the normalized density was calculated by a ratio of the densities for the protein of interest, and the loading control. A one-way ANOVA was used to determine significant differences in means for total GSDMD protein and cleaved GSDMD. No significant differences exist in cleaved GSDMD, but Tukey’s HSD confirmed significant differences for total GSDMD protein levels (*p* < 0.05, *n* = 3).

**Figure 3:**
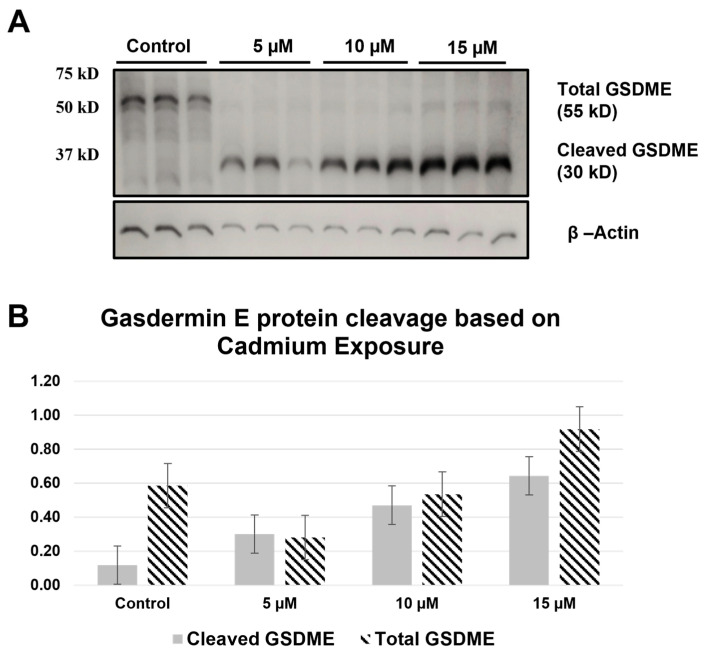
Western blot analysis of gasdermin E. (**A**) A total of 10 μg of whole cell lysate was separated on 10% SDS-PAGE. The blot was probed with GSDME antibody. The blot was developed with ECL. β-Actin was used as a loading control. (**B**) Band intensity was determined using ImageJ, and the normalized density was calculated by a ratio of the densities for the protein of interest and the loading control. A one-way ANOVA was used to determine any significant differences in means for total GSDME protein and cleaved GSDME. No significant differences were found in total or cleaved GSDME (*p* > 0.05, *n* = 3).

**Figure 4:**
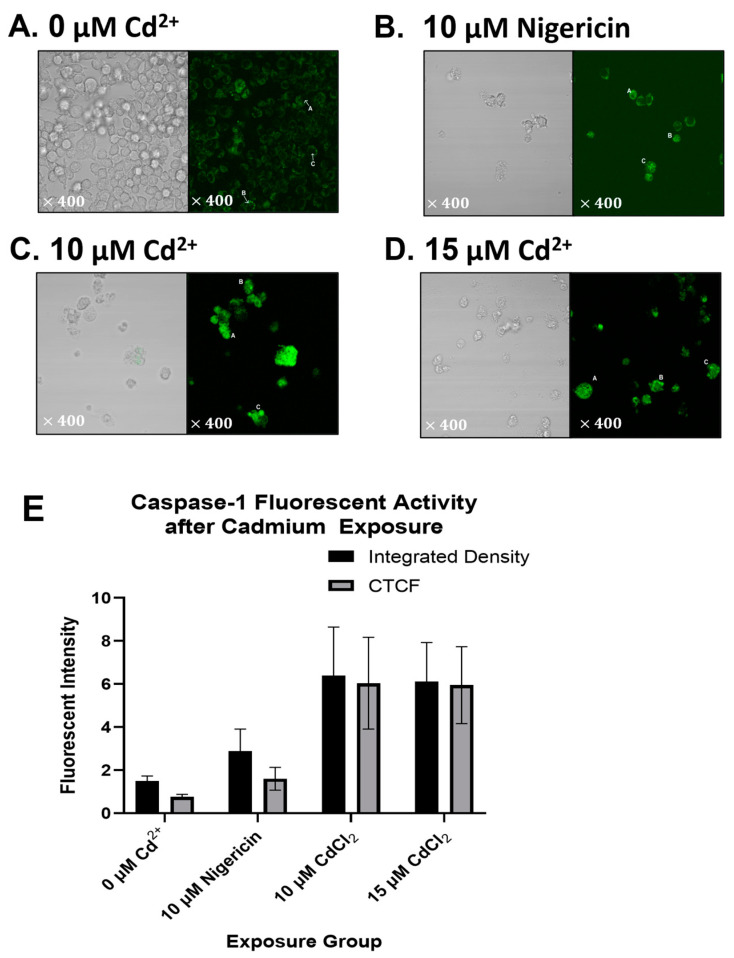
Caspase-1 activation in Cd^2+^-exposed cells. (**A**–**D**) RAW264.7 monocytes were exposed to varying concentrations of CdCl_2_ (0 μM, 10 μM,15 μM) for 24 h, as well as a positive control group (10 μM nigericin). Cells were stained with FAM-FLICA according to the manufacturer’s protocol (Immunochemistry Technologies). Images of cells were taken under brightfield and, after exposure, to fluorescence (400× magnification). (**A**) The control group has low intensity of green coloring, which indicates a lack of caspase-1 activity. (**B**) Cells in the positive control group show intensity in cells undergoing caspase-1-activated inflammatory cell death. (**C**,**D**) The 10 μM and 15 μM CdCl_2_ groups, respectively, show an intense green color, which indicates activation of caspase-1 and inflammatory cell death in these cells. (**A**–**D**) Brightfield images (400× magnification) show a trend of decreasing cell number and altered morphology as Cd^2+^ concentration increases. Data shown represent a good visual of the effects of Cd^2+^ on RAW264.7 monocytes and suggest caspase-1 activation results from this exposure. More experiments should be conducted to draw a definitive conclusion. (**E**) ImageJ was used to measure the intensity of color output in cells (cells labeled “A”, “B”, and “C”, for each group) (**A**–**D**). These intensities were balanced by the area of selection to achieve an integrated density measure. Integrated densities were averaged and balanced against the background to obtain the cell-to-cell fluorescence (CTCF) of each experimental group. The 10 μM and 15 μM Cd^2+^ exposure groups showed higher CTCF and integrated density values, which supports caspase-1 activation when compared to the control group. *n* = 1 experiments were conducted, which accounts for the moderately high standard error of means. More data are being collected to determine if caspase-1 activation is significantly higher than the controls.

Total GSDMD protein decreased as the concentration of CdCl_2_ increased (Figure 2A). This decrease was shown to be significant between controls and both the 10 µM and 15 µM exposure groups (*p* < 0.001, Figure 2B). Furthermore, a significant decrease was observed between the 5 µM and 10 µM as well as the 10 µM and 15 µM exposure groups (*p* < 0.05, *p* < 0.01, respectively. The cleaved GSDMD protein appears in the higher CdCl_2_ exposures (10 µM and 15 µM) but not in the control and 5 µM (Figure 2A). However, one-way ANOVA revealed no significant differences in the mean densities for these bands. GSDME expression showed a similar cleavage pattern to GSDMD, in which the total protein was expressed in control groups with cleavage increasing as CdCl_2_ concentration increased (Figure 3A). However, statistical analysis across triplicates revealed no significant changes between any of the groups for total and cleaved GSDME protein (Figure 3B). As such, Western blot data for GSDME and GSDMD support CdCl_2_ exposure inducing lytic cell death and a release of inflammatory Cytokines in RAW264.7 macrophages. For future experimentation, nigericin will be used as a positive control for Western blots due to its successful caspase-1 activation, which directly leads to gasdermin cleavage during pyroptosis (Figure 4). 

### 2.3. Caspase-1 Activity Increases with Cd^2+^ Exposure 

Caspase-1 has important catalytic activity during pyroptosis; thus, activation of caspase-1 during Cd^2+^ exposure would indicate that pyroptosis is occurring in the RAW264.7 cells. On the light images of the different cells, it is clear from the number of visible cells that a significant portion had died (Figure 4A–D). Likewise, the shape of cells in the control exposure is nicely rounded and has distinct membranes surrounding them. The few cells in the 10 µM and 15 µM exposures have cells that are actively lysing, blebbing, and overall are in a deteriorated state (Figure 4C,D). Under fluorescence, it is clear why these distinct morphological differences exist. The cells exposed to Cd^2+^ show a bright green color, indicating active caspase-1 (Figure 4C,D). Based on quantitation in ImageJ, the cells exposed to Cd^2+^ show more cell-to-cell fluorescence (CTCF) than the positive control nigericin (Figure 4E).

## 3. Discussion 

### 3.1. Extending Understanding of Cd^2+^-Mediated Immune Dysregulation

There is existing knowledge of inflammatory cell death in RAW264.7 macrophages, notably in response to lipopolysaccharide (LPS), which is a sufficient increase in the expression of GSDMD and activates cleavage to create the active N-terminal GSDMD [36,37]. Additional studies in this cell line have revealed insights about the apoptotic mechanism following Cd^2+^ exposure [38,39,40]. However, no studies to date have implicated pyroptosis in this cell line during Cd^2+^ toxicity. One research group in 2016 showed that the mRNA levels of IL-1β decreased with higher Cd^2+^ concentration over 6 h and 24 h [41]. This conclusion argues against the activation of pyroptosis during Cd toxicity, as the release of IL-1β is a hallmark feature of this pathway. However, four out of the five exposure concentrations [0 µM, 0.1 µM, 0.3 µM, and 1 µM] did not significantly reduce cell viability, with one exception [3 µM] [41]. Regardless of the transcriptional regulation of cytokines during Cd^2+^ toxicity, the data showed significant cleavage of GSDMD. 

Additionally, the data support the cleavage of GSDME and activation of Caspase-1. The activation of these key players in pyroptosis provides strong support that pyroptosis is activated during Cd^2+^ toxicity. This study serves as a foundation for a larger investigation of Cd^2+^ toxicity in PANoptosis. Further analyses of downstream mediators, such as cytokines in serum and lysates, need to be performed to confirm the latter effects of Cd^2+^. A better understanding of these mechanisms on a cellular level will help to define potential therapeutic targets that will have functional benefits for the body, for example, in the immune system. It has been well established that the NOD-like receptor protein 3 (NLRP3) inflammasome is a master switch for inflammation and pyroptosis. Furthermore, Cd^2+^ has been shown to induce NLRP3 inflammasome and drive inflammatory cell death. There are a multitude of intrinsic and extrinsic stimuli that activate NLRP3, and one of these is the DNA sensing Z-DNA binding protein 1 (ZBP1) [42]. The ability of ZBP1 to bind viral Z-DNAs and Z-RNAs is rather unique [43]. It allows ZBP1 to activate NLRP3 [44], AIM2 [45], receptor-interacting protein kinase 3 (RIPK3) [46], and other inflammatory responses in the cell. The protein domain that is most vital to the function of ZBP1 is its Zα domain, which is shared with another mammalian protein, adenosine, acting on RNA 1 (ADAR1). ADAR1 is an RNA-editing enzyme that facilitates adenosine-toinosine point mutations and is responsible for increasing the diversity of the transcriptome during viral infection. These proteins have also been identified in the mechanistic progression of PANoptosis [42,45,47]. The pathogenic consequences of disease pathology following Cd^2+^ exposure is similarly found in the IFN response [48].

### 3.2. Cd^2+^ Toxicity in Immune Function Remains Largely Unknown

The activation of pyroptosis provides an understanding of the immune dysregulation in disease states associated with prolonged Cd^2+^ exposure. Many recent reports of Cd^2+^ toxicity use in vitro duck models show the activation of pyroptosis [28,49,50]. There are also several studies that have characterized this pyroptotic mechanism of Cd^2+^ toxicity in human renal tubular epithelial cells, triple-negative breast cancer cells, and human umbilical vein endothelial [51,52]. While these studies do not improve knowledge of cadmium’s effects on the innate immune system, they do contribute to an understanding of structural dysregulation leading to reduced function of the affected organ. However, due to the inflammatory nature of Cd^2+^-mediated chronic diseases, it is imperative that further investigation needs to focus on the key regulators of inflammation in the body, macrophages. By identifying the checkpoints of inflammatory cell death during Cd^2+^ toxicity, therapeutic targets may be revealed that can effectively shut down these cell death responses. If macrophages could potentially resist this Cd^2+^-mediated inflammatory cell death, they would be better able to perform their phagocytic activity and clear damaged structural cells. 

### 3.3. In Vivo Studies May Reveal Functional Outputs 

The model of exposure created and used is similar to other in vitro models of Cd^2+^ toxicity and its effects on immune dysregulation [39,53,54,55]. Moreover, the Occupational Health and Safety Association (OSHA) sets a concentration of ~44 µM Cd^2+^ in blood as the threshold for medical evacuation from employment [56]. This value is far higher than the exposure model, but the actual level of Cd^2+^ in cigarette smoke is about 1–2 µg, and the amount that enters the cell is likely to be even less. Future investigations will use in vivo models to better model the physiological response to Cd toxicity either nebulized Cd^2+^ or the cigarette smoke exposure (CSE) method. 

### 3.4. Limitations and Alternative Strategies

In its current state, this study reveals only preliminary insights into the role of Cd^2+^ in PANoptosis and serves as a pilot study to test the feasibility of an in vitro Cd^2+^ exposure model. Cd^2+^ is a very prevalent toxin in cigarettes, thus implicating it in a multitude of respiratory diseases. However, the exposure route in this study is acute Cd^2+^ toxicity. Future work will utilize a longer-term exposure route to more accurately model the chronic phenotypic outcomes of Cd^2+^-induced airway disease. Additionally, the Caspase-1 Activity Assay was only conducted once. For a statistical conclusion to be drawn, this assay will need to be performed at least two more times to obtain *n* = 3. A rescue experiment with GSDME/D inhibitors/siRNA would provide further evidence on interactions between Cd^2+^ and gasdermins. Lastly, the gasdermin proteins are primarily involved in pyroptosis, which represents just one aspect of PANoptosis. Future studies should investigate other key players across all the pathways under PANoptosis, including other caspases, annexin V, and receptor-interacting protein kinase 3 (RIPK3), to name a few. 

### 3.5. Therapeutic Promise in Ameliorating Cd^2+^ Burden 

Future endeavours stemming from this research may include the exploration of cancer elimination using gasdermin proteins. As previously mentioned in the overview of mechanisms resulting in cell death, gasdermin proteins possess properties that induce pyroptosis. The potential lies in targeting cancer cells and inducing pyroptosis, thus killing those cells and releasing their contents while producing an immune inflammatory response. Problems arise when gasdermin proteins also affect healthy somatic cells. There needs to be further research looking for certain indicators of specificity amongst these proteins and the cells that they target. Other investigations need to find checkpoints further upstream of GSDMD and GSDMDE. Some companies have developed inhibitors for some of the inflammatory protein complexes that act as sensors to initiate inflammatory cell death. One of these, NOD-like receptor protein 3 (NLRP3), is involved in pyroptosis and forms a complex with other cell death proteins, including caspase-1.

## 4. Materials and Methods 

### 4.1. Cell Culture, Cadmium Exposure, and Protein Extraction 

RAW 264.7 macrophages (ATCC, TIB-71™) were grown in DMEM with 10% FBS and antibiotic/antimicrobial solution. After culturing in T-75 flasks, 1.5 million cells were seeded in 12,100 mm dishes. At 90% confluency, cells were plated onto 12,100 mm petri dishes. For 24 h, a control group of untreated RAW cells (0 μM CdCl_2_) and RAW cells exposed to three concentrations of solid CdCl_2_ (Sigma Aldrich, St. Louis, MO, USA, CAS: 10108-64-2) (5 µM, 10 µM, 15 µM) were incubated at 37 °C at 5% CO_2_. After treatment duration, cells were collected and pelleted for extraction. Protein lysis buffer with 100X Protease Inhibitor (Sigma Aldrich, St. Louis, MO, USA, P8340-5ML) and 100X Phosphatase Inhibitor (MilliporeSigma, Burlington, MA, USA, P0044-5ML) was diluted to 1X in solution with the cell pellets. Protein lysates were sonicated for 3 min with 15 s cycles on and off. Then, the lysates were spun at 14,000 rpm at 4 °C for 15 min. The supernatant was collected and transferred to low-binding Eppendorf tubes for storage at –70 °C. 

### 4.2. Cytotoxicity Assay 

RAW264.7 cells were seeded in a 96-well plate at a density of 1 × 10^3^ cells/well in 100 µL of constituted growth media (Dulbecco’s Modification of Eagles Medium (DMEM), Fisher Scientific, USA, 10% Fetal Bovine Serum (FBS), 1X Antibiotic/Antimycotic Solution. The plate was incubated for 24 h at 37 °C 5% CO_2_. Cells were then exposed to varying concentrations of CdCl_2_ (0–50 µM) for 24 h. A total of 10 µL of the CCK-8 solution was added to each well and incubated according to the manufacturer’s protocol. After incubation, the plate was gently mixed and read at an absorbance of 450 nm. Subsequent calculations of cell viability were conducted according to the manufacturer (GLPBio, Canada, GK10001). Three separate experiments were performed for statistical analyses to be performed.

### 4.3. SDS PAGE and Western Blot Analysis 

Gels were cast using standard recipes for SDS polyacrylamide gels: 10% gels were used for these experiments. A total of 10 µg of protein lysate was loaded into each well, in addition to 4X Laemmli dye and lysis buffer solution. The gels were run at ~70 V until stacking was complete and then at ~105 V until the marker was clearly spaced and wells had run almost off the gel. Gels were transferred at 30 V overnight to a PVDF membrane in a 1X transfer buffer. Membranes were blocked with 5% skim milk/TBST. After probing with primary antibodies (GSDMD Abcam 1:1000, GSDME Abcam 1:1000, Cambridge, UK) membranes were probed with secondary antibodies (JAX labs, Boston, MA, USA, AntiRabbit 1:10,000). Using ECL (Thermofisher, USA, PI32106) and Femto (Thermofisher, USA, 34094) reagent kits, blots were developed in a BioRad ChemiDoc™ MPimaging system. Images were quantitated via ImageJ (version 1.54j), and subsequent band intensities were calculated based on a ratio of the band of interest to the loading control (β-Actin).

### 4.4. Caspase-1 Activation Kit

To determine the level of caspase-1 activation in RAW264.7 macrophages, the FAM-FLICA Caspase-1 Assay Kit (Immunochemistry Technologies, Davis, CA, USA) was used. Cells were plated to a 4-compartment petri dish (Celltreat Scientific, Pepperrell, MA, USA) at the seeding density recommended by ThermoFisher Scientific (USA) for 100 mm dishes (total divided by 4, per compartment). Cells were exposed to CdCl_2_ for 24 h, as in the initial experiments. Compartment 1 was the control group, exposed to no CdCl_2_. Compartment 2 was exposed to 10 μM Nigericin. Nigericin was used as a positive control as it induces the cleavage of GSDMD while having no direct relationship to GSDME expression. Compartment 3 was exposed to 10 μM of CdCl_2_, and Compartment 4 to 15 μM CdCl_2_. After the 24 h exposure period, cells were stained with FAM-FLICA reagent according to the manufacturer’s instructions (Immunochemistry Technologies) and imaged under brightfield and fluorescent microscopy (Olympus IX51, Olympus Life Science, Tokyo, Japan). Microscopy was quantified using ImageJ (version 1.54j) for integrated density and cell-to-cell fluorescence.

### 4.5. Statistical Analysis 

Data represent mean values ± SEM. For Western blot data (Figure 2 and Figure 3), data are representative of the average normalized density for each experimental group. Normalized densities were calculated by taking a ratio of the protein of interest to a loading control (β-Actin). A one-way ANOVA was conducted using R Studio (version 2024.04.0+735) to quantify differences between cadmiumtreated cells compared with control cells in all cases. Post hoc testing using Tukey’s HSD (R Studio, version 2024.04.0+735) was used to identify which pairwise comparisons were significant, if any. Significance was set at *p* < 0.05. All significant data were confirmed by three separate experiments.

## Data Availability

The original contributions presented in the study are included in the article. Further inquiries can be directed to the corresponding author.
